# Supplementary material for: A survey for characterizing details of fall events experienced by lower limb prosthesis users
Source: PLoS One. 2022 Jul 28;17(7):e0272082. doi: 10.1371/journal.pone.0272082 (PMC9333270; doi:10.1371/journal.pone.0272082)
Supplement: S1 Appendix — (DOCX) [file pone.0272082.s001.docx]

**Fall Survey for People with Lower Limb Amputation**

**Cognitive Interview Guide**

**Questions for All Cognitive Interviews**

**Instructions**

*Did you read the instructions?*

⬜ Yes ⬜ No

**If NO:**  *That’s ok. I will have you look at them in just a moment. Before you do, can you tell me what you thought about when you read the words “fall” and “near-fall” in the survey? What is a fall? A near-fall?*

**Fall:**

**Near-fall:**

*How would you differentiate between a fall and near-fall?*

*Ok, great, thank you. Now, would you please read the instructions for me?*

[proceed to “**If YES**”]

**If YES:** *Were any of the survey instructions confusing? If so, which part(s)?*

*Would you recommend any changes to the instructions to make them easier to understand?*

*When you read our definition of a “fall” and a “near-fall,” was it clear what we wanted you to think about? What did you think we were asking about?*

*Were there any parts of our definitions that were confusing? Would you recommend using words like “accidental”?*

*Based on the definitions we provide, can you tell how a “fall” and “near-fall” are different?*

**Number of fall or near-fall experiences**

*In the section on fall or near-fall experiences, was it clear that we wanted you to think about falls and near-falls that occurred within the past 12 months? Did you have any trouble answering the survey based on this timeframe? If so, can you describe what was difficult?*

*What did you check where we asked “In the past 12 months, have you experienced a fall where your body accidentally landed on the ground or floor”?*

⬜ Yes ⬜ No ⬜ Neither

*What did you enter above the “number of falls”?*

____________

*What did you check where we asked “In the past 12 months, have you experienced a near-fall where you caught yourself or recovered your balance before your body landed on the ground or floor”?*

⬜ Yes ⬜ No ⬜ Neither

*What did you enter above the “number of near-falls”?*

____________

**If the participant answered only the fall OR near-fall question, or answered “yes,” but did not include a number:**

*How could we change the survey, if we wanted someone to be sure to answer the question both about “falls” and “near-falls”? Was there a reason that you didn’t answer both questions?*

*How could we change the survey, if we wanted someone to be sure to answer the question about falls/near-falls AND enter the number of falls/near-falls?*

**Stopping the survey**

*Under what situations would someone stop answering questions on the survey?*

*How could we change the survey to make it clearer when we want people to stop?*

[provide detail about stopping, as needed]

**Most recent fall or near-fall**

*How did you answer question #3 “Was your most recent event a fall or near-fall”?*

⬜ Fall ⬜ Near-fall ⬜ Neither

*How would someone answer this question if they had experienced their latest fall 1 month ago, and their latest near-fall last week?*

⬜ Fall ⬜ Near-fall ⬜ Unknown

*How would someone answer this question if they had not experienced a fall in the past 12 months, and had a near-fall 1 month ago?*

⬜ Fall ⬜ Near-fall ⬜ Unknown

*How did you answer question #4 “What was the date of your most recent fall or near-fall?*

Month: ___________ Year: ________________

*Is it hard to remember the date of a fall or near-fall event? If so, can you think of a better way for us to ask someone about when his or her last fall or near-fall occurred?*

**Significance of most recent fall or near-fall**

*What does the term “significant” mean to you?*

*What makes a fall “significant” to you?*

Are there other words or terms we could use to ask someone if a loss of balance or fall was personally “significant”?

**Final summary question**

*What other aspects of balance and falls do you think should be included in the survey? What kinds of things are important for us to ask about if we want to learn more about falls?*

*Do you have any other suggestions for things we should include in this survey?*

**Questions for Cognitive Interview Group 1**

**Assistive Device**

*How did you respond to question #6, “Were you using any of the following assistive devices at the time of your fall or near fall?”*

⬜ Cane ⬜ Crutch ⬜ Walker ⬜ Wheelchair ⬜ None ⬜ Do not remember

*Are there any other types of assistive devices that you use that were not listed?*

*Is it possible to be using more than one form of assistive device at the time of a fall?*

*Have you ever fallen while using that device?*

⬜ Yes ⬜ No

**Time of Day**

*How did you respond to question #12, “What time of day was it?”*

⬜ Morning (6am-12pm) ⬜ Afternoon (12pm-6pm)

⬜ Evening (6pm-12am) ⬜ Night (12am-6am)

⬜ Do not remember

*How did you decide which answer to choose?*

*Did you use the description (eg, “morning” or “evening”) and/or the time (eg, “6am-12pm”) to select your answer?*

*Would you change how the time of day was divided up? If so, how?*

**Situations**

*How did you respond to question #18 “Did any of the following occur when you fell or nearly fell?”*

⬜ You misplaced a step ⬜ Your feet slipped from underneath you

⬜ You were avoiding an obstacle or object ⬜ You caught your leg or foot on something

⬜ You were bumped, pushed, or pulled ⬜ You were moving in a crowded space

⬜ Your feet were too close together ⬜ You were walking on a narrow surface

⬜ You were carrying an object ⬜ Your vision was obstructed

⬜ The floor or ground moved unexpectedly ⬜ The floor or ground changed levels

⬜ Your prosthesis broke ⬜ Your prosthesis moved unexpectedly

⬜ Your socket was loose ⬜ Your prosthesis was not on properly

⬜ Your assistive device broke ⬜ Your assistive device moved unexpectedly

⬜ Do not remember ⬜ None of these apply

*Can you think of a better way we could ask this question?*

*Do the answers represent realistic scenarios that might cause someone to lose their balance?*

*If not, which of the scenarios were unrealistic?*

*Did any of the answers seem repetitive? If so, which ones?*

*Did you have any difficulty in selecting your answer(s)? If so, why?*

*Was it clear that you could choose multiple answers? Can you imagine a situation where multiple answers might apply?*

**Possible answers**

*Now I would like to ask you questions about each of the possible answers. Even though you may not have checked a box for the last fall or near-fall you have experienced, I’d like you to try to think about situations where the scenarios might cause someone to lose their balance.*

**Misplaced step**

*What would be an example of misplacing a step?*

*Are you more or less likely to misplace a step with your prosthetic leg? Why?*

*Are there certain situations where you are more likely to misplace a step?*

*Can you describe how someone may lose his or her balance by misplacing a step?*

**Feet slipping**

*Can you describe a situation where your feet may slip from underneath you and cause you to lose your balance?*

*What direction do your feet usually go relative to your body when you slip?*

**Obstacles or objects**

*What obstacles or objects do you avoid because they might cause you to lose your balance?*

*Is there a difference between an obstacle and an object? If so, what is the difference?*

*How would you move your body in order to avoid an obstacle or object?*

*Can you describe a situation where avoiding an obstacle or object may cause you to lose your balance or fall?*

**Catching a foot**

*When you think about catching your leg or foot on something what comes to mind?*

*What specific things might you catch your leg or foot on that would cause you to lose your balance or fall?*

**Bumped, pushed, pulled**

*Do you think being bumped, pushed, or pulled could cause you to lose your balance?*

*Would getting bumped, pushed, or pulled affect balance in different ways? How?*

*Do you think that one causes people with lower limb amputation to lose their balance more than the others?*

*What do you do to maintain your balance when you are bumped, pushed, or pulled?*

**Crowded space**

*How would you describe a crowded space?*

*Do you avoid crowded spaces, or do you tend to find yourself dealing with them frequently?*

*Can you describe a specific situation where you had to move in a crowded space?*

*Have you ever felt as though moving in a crowded space might cause you to lose your balance and/or fall?*

*Do you move differently when you are in a crowded space?*

**Feet close together**

*Do you think the placement or positioning of your feet affects your balance or likelihood of falling?*

*Can you describe a situation where you had to place your feet closer together?*

*Are there situations or environment where you find yourself having to place your feet closer together than you normally would? Does this make it more difficult to maintain your balance?*

*Do you do anything different in order to maintain your balance when you have to put your feet close together?*

**Narrow surfaces**

*Can you give me some examples of narrow surfaces that people have to walk or step on?*

*Can you describe situations where walking on a narrow surface could cause someone to lose their balance?*

*How “narrow” does a surface have to be to make someone lose their balance?*

*Would you change how you walk or stand on a narrow surface to avoid a loss of balance or fall?*

**Carrying an object**

*Are you more likely to lose your balance or fall while carrying an object? Why or why not?*

*What kind of objects might increase the chance you would lose your balance?*

*How might you change how you carry an object so that your balance is not affected?*

**Obstructed vision**

*When you think of your vision being obstructed what comes to mind?*

*What are some reasons you think someone’s vision may be obstructed?*

*Would having your vision obstructed affect your balance? Why?*

**Floor or ground moving**

*What are some examples of the floor or ground moving unexpectedly?*

*What types of unexpected floor or ground movement would make it difficult to maintain your balance?*

*How would you respond to unexpected movements of the floor or ground in order to maintain your balance and avoid a fall?*

*Do the words “ground” and “floor” mean the same thing to you? If not, how are they different?*

**Floor or ground changing levels**

*Can you describe any examples where you felt that the floor or ground changed levels on you?*

*What kinds of changes in the level of the floor or ground might cause you to lose your balance and/or fall?*

*What do you do when the floor or ground change levels to maintain your balance?*

**Prosthesis break**

*Have you ever had your prosthesis break while using it? Please describe what happened.*

*Can you imagine a situation where a prosthesis breaking might lead to a fall?*

**Prosthesis move unexpectedly**

*Have you ever had your prosthesis move unexpectedly? What happened?*

*What types of prosthesis movements would you describe as “unexpected”?*

*Are there specific types of unexpected prosthesis movements that may lead to a loss of balance or fall?*

**Loose socket**

*What does the phrase “a loose socket” mean to you?*

*Can you describe what things might cause your socket to feel loose?*

*Do you move differently when your socket is loose? How so?*

*Does a loose socket affect your balance? If so, how?*

**Prosthesis not on properly**

*Have you ever used your prosthesis when it wasn’t on properly?*

*What does it feel like when your prosthesis is not on properly?*

*What things affect whether your prosthesis is on properly or not?*

*Can you describe a situation when your prosthesis not being on properly may cause you to lose your balance and/or fall?*

**Assistive device move unexpectedly**

*What types of things would you include under “assistive devices”?*

*Do you ever experience your assistive device moving unexpectedly? How?*

*How could an assistive device move unexpectedly and cause a fall?*

**Missing work**

*Now, I’d like to ask you about question #27 related to missing work. How did you answer the question “How much time did you miss from work because of your fall or near-fall?”*

⬜ Less than 1 day ⬜ 1 day

⬜ 2-3 days ⬜ More than 3 days

⬜ None of these apply ⬜ Do not remember

*What does the term “work” mean to you?*

*Are the answers we present realistic for someone who might miss work due to a fall-related injury? If not, how would you suggest we change them?*

*Would you expect a fall or near-fall to lead to someone missing work? If so, for how long?*

**Significance of most recent fall or near-fall**

*What does the term “significant” mean to you?*

*What makes a fall “significant” to you?*

Are there other words or terms we could use to ask someone if a loss of balance or fall was personally “significant”?

**Questions for Cognitive Interview Group 2**

**Physical assistance prior to event**

*How did you respond to question #8, “Was someone physically assisting you just before you lost your balance?” (Choose the best answer)*

⬜ Yes ⬜ No ⬜ Do not remember

*What does it mean to give or receive “physical assistance”?*

*Was it clear that you were being asked about physical assistance you might have been receiving just before the fall or near-fall? If not, how could we revise this question to make that clear?*

**Physical assistance to avoid a fall**

*How did you respond to question #9, “Did someone physically assist you once you started to lose your balance?*

⬜ Yes ⬜ No ⬜ Do not remember

*Can you describe how someone nearby might help you when you start to fall?*

*Imagine that you lost your balance while out walking with a friend and, to keep from falling you reached out and grabbed him or her. Which response would you choose if this situation had occurred?*

⬜ Yes ⬜ No ⬜ Do not remember

**If YES:** *How could we modify the question so that it is clear we are only asking about situations where someone else reaches out to help you (and not the other way around)?*

**Lighting**

*How did you respond to question #13, “What was the lighting like?” (Choose the best answer)*

⬜ Well lit ⬜ Poorly lit ⬜ Do not remember

*What kinds of locations are typically well lit?*

*What kinds of locations typically are poorly lit?*

*How important is good lighting to maintaining your balance?*

**Activities**

*Now, I’d like to ask you about the question related to activities you may have been engaged in at the time of the fall or near-fall.*

*How did you respond to question #17, “Were you doing any of the following?”*

⬜ Standing up from a seated position ⬜ Sitting down from a standing position

⬜ Turning towards your left side ⬜ Turning towards your right side

⬜ Starting to move ⬜ Coming to a stop

⬜ Speeding up ⬜ Slowing down

⬜ Do not remember ⬜ None of these apply

*Was it clear that you could choose multiple answers?*

*Was the layout of the answers (i.e., vertical vs. side-by-side) clear and easy to follow? Would you prefer a different layout? What suggestions do you have for presenting survey answers?*

*Do the answers represent realistic activities that might cause someone to lose their balance? If not, which of the answers were unrealistic?*

*Are there other activities that we should list here?*

*Did any of the answers seem redundant? If so, which ones?*

**Possible answers**

*Now I would like to ask you questions about each of the possible answers. Even though you may not have checked a box for the last fall or near-fall you have experienced, I’d like you to try to think about situations where performing these activities might cause someone to lose their balance.*

**Standing up**

*What part of standing up from a seated position is most likely to cause someone to lose his or her balance?*

*How does standing up from a chair differ for people who use a lower limb prosthesis?*

**Sitting down**

*Can you describe how someone may lose his or her balance while sitting down?*

*How might someone who wears a prosthesis sit down so that they don’t fall?*

**Turning toward your right or left side**

*Can you describe how someone may lose his or her balance when turning?*

*Is turning more or less challenging for people with a prosthesis? Why?*

*What is different about turning to your right or your left side? Does turning one direction challenge your balance more than the other?*

**Starting to move**

*What does the phrase “starting to move” mean to you?*

*Can you describe what you imagine when you think about starting to move?*

*How might starting to move cause someone to lose his or her balance?*

**Coming to a stop**

*Can you describe a situation where coming to a stop might cause someone to lose their balance?*

*What direction do you think someone is likely to fall when “coming to a stop”?*

*How does coming to a stop on your prosthetic or non-prosthetic leg affect your balance?*

**Speeding up**

*How does speeding up affect someone’s balance?*

*When you thought about speeding up, how quickly did you picture yourself moving?*

*Do you think someone with a prosthesis is more likely to lose his or her balance while speeding up or slowing down? Why?*

**Slowing down**

*Can you describe for me the last time you had to slow down quickly?*

*What are some examples of situations where slowing down might throw someone off balance?*

**Fall direction**

*Now, I’d like to ask you about the question related to directions of a fall or near-fall.*

*How did you answer question #20, “In what direction did you fall or nearly fall?”*

⬜ Forward ⬜ Backward

⬜ Left ⬜ Right

⬜ Straight down ⬜ Do not remember

⬜ None of these apply

*When you think about the term “direction” what does it mean to you?*

*Imagine you fell diagonally (for example, not straight forward and not directly to your left), how would you answer the question?*

**Possible answers**

*Now I would like to ask you questions about each of the possible answers. Even though you may not have checked a box for the last fall or near-fall you have experienced, we still want to get your opinions on each of the possible answers.*

**Forward**

*Can you describe what a forward fall would look like?*

*What might cause someone to lose his or her balance and fall forwards?*

**Backwards**

*What does a backward fall look like?*

*Can you think of a situation where someone would lose his or her balance and fall backwards?*

**To the left/to the right**

*Can you describe a situation where someone would fall to his or her left? To their right?*

*What kinds of things might cause a loss of balance to the right or left, but not forward or backward?*

**Straight down**

*What does it mean to fall “straight down”? Can you describe how this might occur?*

*What parts of the body might you expect to hit the ground when someone falls straight down?*

**Injury**

*Now, I’d like to ask you about the question related to injuries after a fall or near-fall.*

*How did you respond to question #24, “Did you experience an injury because of your fall or near-fall?”*

⬜ Bruise ⬜ Cut or scrape

⬜ Pain or soreness ⬜ Swelling

⬜ Pulled muscle ⬜ Torn tendon or ligament

⬜ Fracture or broken bone ⬜ Joint dislocation

⬜ Internal injury ⬜ Concussion or head injury

⬜ Did not experience an injury ⬜ Do not remember

⬜ None of these apply

*Was there an injury listed that you would* ***not*** *expect someone who falls to experience? If so, which one(s)?*

*Is there any other type of injury that could come from a fall that* ***was not included in this list****? If so, please describe.*

*Were there any answers (types of injuries) listed that were confusing?*

**Questions for Cognitive Interview Group 3**

**Accompanied or unaccompanied fall / near-fall**

*How did you respond to question #7, “Were you alone or with others?”*

⬜ Alone ⬜ With Others ⬜ Do not remember

*What does “with others” mean to you (i.e., 1 other person? A group?)*

*How does being around others affect your balance?*

*How close to you (proximity) does someone have to be to you in order for them to be considered “with you”? Same room? Arms length?*

**Familiarity of location**

*How did you respond to question #10, “Were you in a familiar or unfamiliar location?”*

⬜ Familiar ⬜ Unfamiliar ⬜ Do not remember

*What makes a location familiar or unfamiliar?*

*How does the familiarity of a location affect your balance or likelihood of falling?*

**Situations**

*How did you respond to question #16 “Were you doing any of the following?”*

⬜ Going up stairs ⬜ Going down stairs

⬜ Going up a hill, ramp, or incline ⬜ Going down a hill, ramp, or incline

⬜ Stepping up onto a surface ⬜ Stepping down from a surface

⬜ Do not remember ⬜ None of these apply

*Can you think of a better way we could ask this question?*

*Do the answers represent realistic scenarios where someone might lose their balance?*

*If not, which of the scenarios were unrealistic?*

*Did any of the answers seem repetitive? If so, which ones?*

*Did you have any difficulty in selecting your answer(s)? If so, why?*

*Are there other situations in which you have fallen that we haven’t included?*

*Was it clear that you could choose multiple answers? Can you describe a situation where multiple answers might apply?*

**Possible answers**

*Now I would like to ask you questions about each of the possible answers. Even though you may not have checked a box for the last fall or near-fall you have experienced, I’d like you to try to think about situations where the scenarios might cause someone to lose their balance.*

**Going up / down stairs**

*Does going up or down a flight of stairs affect your balance in different ways? If so, what is the difference?*

*Can you describe how someone may lose his or her balance going up or down a flight of stairs?*

*What about going up or down a flight of stairs poses the greatest challenge to your balance (e.g., stepping up or down with one leg or the other, making sure your toes don’t catch on the step)?*

**Going up / down a hill, ramp, or incline**

*How would you describe the difference between a hill, a ramp, and incline? Are these the same or different?*

*Does going up or down a hill, ramp, or incline affect your balance in different ways? If so, what is the difference?*

*Can you describe how someone may lose his or her balance going up or down a hill, ramp, or incline?*

*What part of going up or down a hill, ramp, or incline poses the greatest challenge to your balance (e.g., moving over one leg or the other, balancing while stepping with one leg)?*

**Stepping up onto / down from a curb**

*Would stepping up onto or down from a curb affect balance in different ways? How?*

*Can you describe how someone may lose his or her balance stepping up onto or down from a curb?*

*Are you more or less likely to step up on to or down from a curb with one leg or the other? Why?*

**Preventing a fall or minimizing risk of injury**

*Now, I’d like to ask you about the question related to preventing the fall or minimizing the risk of injury. How did you answer question #21 “Did you do anything to catch yourself, prevent the fall, or minimize the risk of injury?*

⬜ Reached out to grab someone/something ⬜ Leaned against someone/something

⬜ Skipped or hopped ⬜ Took a big step

⬜ Changed position to limit/avoid impact ⬜ Do not remember

*Are the answers we present realistic for someone who might be trying to prevent a fall or minimize the risk of injury? If not, how would you suggest we change them?*

*When you lose your balance or fall, what things do you do to try and regain your balance?*

*Are there any other strategies you use to regain your balance that we haven’t included?*

*Is there a “safest” way for you to fall?*

**Possible answers**

*Now I would like to ask you questions about each of the possible answers. Even though you may not have checked a box for the last fall or near-fall you have experienced, I’d like you to try to think about situations where the scenarios might cause someone to lose their balance.*

**Reached out to grab someone/something**

*What would be an example of something you might reach out to grab to stop yourself from falling or limit your chances of hurting yourself while you fell?*

*Is reaching out to grab someone or something a realistic way to prevent a fall? To minimize the risk of injury? Why or why not?*

*Can you describe a situation where reaching out to grab someone or something prevented a fall and/or minimized your risk of injury when you fell?*

**Leaned against someone/something**

*What would be an example of something you might lean against when you lose your balance?*

*Is leaning against someone or something an effective way to minimize the risk of injury? Why or why not?*

*Can you describe a situation where leaning against someone or something prevented you from falling? If not, can you imagine a situation where this might happen?*

*Are you more likely to lean against or reach out and grab someone or something in order to prevent a fall and/or minimize the risk of injury?*

*Are there any situations where reaching out to grab someone or something could cause more injury?*

**Skipped or hopped**

*What is the difference between skipping and hopping?*

*Have you ever tried to skip or hop to avoid a fall or minimize the risk of injury? What happened? Did it work?*

*Are you more or less likely to skip or hop on one leg when you lose your balance? Which one and why?*

**Took a big step**

*Can you describe a situation where taking a big step may prevent a fall and/or minimize risk of injury?*

*Are you more or less likely to take a big step with one leg, if you’re trying to regain your balance? Why?*

*Are there certain situations where taking a big step is the best way to prevent a fall?*

**Adjusted or selected a body position to avoid impact?**

*How would you change position to limit or avoid impact you made when you hit the ground?*

*Can you describe a situation where changing position allowed you to avoid a fall? What about a situation where you still fell, but changing your position reduced the impact of the fall?*

*Are there specific areas on your body that better tolerate the force of a fall? Are there areas that you try really hard to avoid hitting?*

**Functional, behavioral, and emotional consequences**

*Now, I’d like to ask you about the question related to whether you have made any changes after a fall or near-fall. How did you answer question #28 “Because of this fall or near-fall have you done any of the following?*

⬜ Changed how you do certain activities ⬜ Required more assistance to perform activities

⬜ Avoided certain activities ⬜ Stopped doing certain activities

⬜ Rested more than usual ⬜ Become more fearful of falling

⬜ Lost confidence in your balance ⬜ Felt embarrassment

⬜ Don’t remember ⬜ None of these apply

*Did any of the answers seem repetitive? If so, which ones?*

*Was it clear that you could choose multiple answers? If not, how could we make it clear that it is OK for respondents to check multiple boxes?*

*Do the answers represent realistic changes someone might make after a fall or near-fall? If not, which of the scenarios were unrealistic?*

*Are there any other changes someone might make or feelings someone might have after a fall that we should include here?*

*Over what period of time were you thinking about as you read this question?*

*(Prompt if needed: day, week, month, longer… shorter?)*

**Possible answers**

*Now I would like to ask you questions about each of the possible answers. Even though you may not have checked a box for the last fall or near-fall you have experienced, I’d like you to try to think about situations where the scenarios might cause someone to lose their balance.*

**Change how you do certain activities**

*When you think about the term “activity” what comes to mind?*

*How might someone perform an activity differently after a fall or near-fall? Can you think of an example or two?*

*Can you think of a situation where someone might change how they do many activities after a fall?*

*What about a situation where they might change just one activity?*

*For how long after a fall or near-fall would you expect someone would change how he or she does any activity? Why do you say that?*

**Required more assistance to perform activities**

*What came to mind when you read the word “assistance”?*

*Does “assistance” include help from a person, help from a device like a cane, or both?*

*Is there a difference between the phrases “required more assistance” and “required assistance”? If so, what is the difference?*

*Can you think of any ways that someone might need “more assistance” after a fall or near-fall?*

*How long after the fall or near-fall do you think someone would need more assistance to perform activities?*

**Avoid certain activities**

*When you think of the term “avoid” what comes to mind?*

*Can you describe a situation where a fall or near-fall might cause someone to avoid certain activities? What activities might that include?*

*When you think about avoiding activities after a fall or near-fall, do you imagine avoiding activities for just a short period of time, or a longer period of time?*

**Stop doing certain activities**

*Would you consider “avoiding certain activities” different from “stopping certain activities”? If so, how?*

*How long would it take someone to “avoid an activity” for you to decide they had “stopped doing that activity?*

*Is “avoided certain activities” and “stopped doing certain activities” different? If yes, how?*

*Can you describe a situation where a fall or near-fall might cause someone to stop doing an activity? What activities might that include?*

**Rested more than usual**

*When someone says they “rested”, what does that mean to you? What does “resting” after a fall or near-fall involve?*

*What is a “usual” amount of rest, and what types or amount of rest would be considered “more than usual”?*

*Can you describe a situation where someone might need to rest more than usual after a fall or near-fall?*

**Become more afraid of falling**

*What does it mean to be “afraid of falling”?*

*Why might someone be afraid of falling?*

*Is it possible to be “concerned” or “worried” about falling, but not be “afraid”?*

*How would a fall or near-fall increase someone’s fear of falling?*

**Lost confidence in your balance**

*What does “confidence in your balance” mean to you?*

*Can you think of a time when you lost confidence in your balance? Can you describe it?*

*What changes when someone loses confidence in his or her balance?*

*How does a loss of confidence in your balance differ from a fear of falling?*

**Felt embarrassment**

*What does it mean to “be embarrassed”?*

*What aspects of losing your balance or falling might be embarrassing?*

*How long does any embarrassment related to a fall last?*

**Questions for Cognitive Interview Group 4**

**Activities at the time of the event**

*Now, I’d like to ask you about the question related to activities you may have been engaged in at the time of the fall or near-fall.*

*How did you respond to question #15, “What were you doing?”*

⬜ Sitting still ⬜ Standing still

⬜ Stepping backwards ⬜ Stepping forwards

⬜ Stepping towards your left side ⬜ Stepping towards your right side

⬜ Reaching forwards ⬜ Reaching backwards

⬜ Reaching towards your left side ⬜ Reaching towards your right side

⬜ Bending over ⬜ Walking

⬜ Running ⬜ Participating in sports or exercise

⬜ Do not remember ⬜ None of these apply

*Was it clear that you could choose multiple answers?*

*Do the answers represent activities during which someone may lose their balance and/or fall? Are there any activities listed that we should remove?*

*Have we missed any activities that we should add to the list?*

*Do any of the answers seem repetitive? If so, which ones?*

**Possible answers**

*Now I would like to ask you questions about each of the possible answers. Even though you may not have checked a box, I’d like you to try to think about situations where performing these activities might cause someone to lose their balance or fall.*

**Sitting still**

*How does the surface or object someone is sitting on affect their balance?*

*Can you describe how someone may lose his or her balance while sitting?*

**Standing still**

*When you thought about standing still, what were you picturing? For how long were you standing still?*

*What aspects of standing still do you think pose the greatest challenge to someone’s balance?*

*How does standing still affect someone wearing a prosthesis?*

**Stepping backwards**

*Under what type of situations would you expect someone to have to step backwards?*

*What would you describe as the most challenging part of stepping backwards for people who are wearing a prosthesis?*

*Is stepping backwards more challenging to someone’s balance than stepping in any another direction? If so, why?*

**Stepping forwards**

*How does stepping forward on your left or right leg challenge your balance more?*

*How does stepping forward differ from stepping backwards?*

**Stepping to your left or right side**

*Can you describe how someone may lose his or her balance when stepping to the side?*

*Does stepping to the side create more challenge to someone’s balance for people with a prosthesis? Why?*

**Reaching forwards**

*What things could someone do to maintain his or her balance while reaching forward?*

*Under what circumstances might someone lose their balance and fall when reaching forward?*

**Reaching backwards**

*Can you describe a situation when someone would need to reach backwards and possibly lose their balance?*

*Is it harder or easier to keep your balance when reaching behind you, rather than in front of you? Why?*

*What could you do to maintain your balance if you needed to reach behind you?*

When you think about reaching backwards, what does that movement look like? Can you describe the position of your body?

**Reaching to your left or right side**

*How is your balance affected when you reach to the right? To the left?*

*When reaching to the side, which direction challenges your balance the most? Why?*

*How do you think reaching causes balance problems for people who wear prostheses?*

**Bending over**

*Can you describe a time that you fell or lost your balance when you were bent over?*

*How do you think bending over might cause someone to lose his or her balance?*

**Walking**

*What part of walking is most likely to cause someone to lose his or her balance?*

**Running**

*How does running affect your balance differently than walking?*

*Would you expect a fall to be different when someone is running versus walking? If so, how?*

**Participating in sports or exercise**

*When you thought about “participating in sports* or exercise*” what types of things came to mind?*

*In what types of sports* or exercise *would you expect someone to lose their balance and fall?*

*Are there sports* or exercise *where someone wearing a prosthesis might be more likely to fall? Why?*

**Situations**

*Now, I’d like to ask you about the question related to situations you may have found yourself in at the time of the fall or near-fall.*

*How did you respond to question #19, “Did any of the following contribute to your fall or near-fall?”*

⬜ You were distracted or doing two things at once ⬜ You were in a hurry or in a rush

⬜ You were tired from a lack of sleep ⬜ You were fatigued from activity

⬜ You consumed alcohol or took drugs ⬜ You were on medications

⬜ Do not remember ⬜ None of these apply

*Was the layout of the answers (i.e., vertical vs. side-by-side) clear and easy to follow? Would you prefer a different layout? What suggestions do you have for presenting survey answers?*

*Was there a situation listed that you would* ***not*** *expect to contribute to a loss of balance? If so, which one(s)?*

**Possible answers**

*Now I would like to ask you questions about each of the possible answers. Even though you may not have checked a box for the last fall or near-fall you have experienced, I’d like you to try to think about if and how these situations might cause someone to lose their balance.*

**Distracted**

*What does the word distracted mean to you?*

*How do you think being distracted might affect someone’s balance?*

*Can you describe a time when you lost your balance because you were distracted?*

*Are being distracted and doing two things at once the same thing? Is there a situation where they are different?*

*Does being distracted and doing two things at once have a similar or different effect on someone’s balance? Why?*

**Rush or hurry**

*Does being in a hurry mean the same thing as being in a rush? If not, how are they different? If they are the same, which is the easiest to understand?*

*What types of situations might cause someone to rush or be in a hurry, and cause someone to lose their balance?*

*In what ways could being in a hurry or a rush affect someone’s balance?*

**Tired**

*How does being tired from a lack of sleep affect your balance?*

*Can you describe a time when you were tired and it affected your balance?*

**Fatigued**

*Is being fatigued from activity different than being tired from a lack of sleep? If so, how?*

*How does fatigue from activity affect your balance?*

*Can you remember a time when you experienced a fall or near-fall because you were fatigued from activity? If so, what happened?*

*Are you more concerned about your balance when you are fatigued from activity? If so, why?*

**Alcohol or Drugs**

*What types of things would “alcohol or drugs” include? (e.g., does it include both legal and illegal drugs, beer, wine, and liquor?)*

*Do you think that consuming alcohol or drugs could affect someone’s balance? If so, how?*

**Medication**

*What does “medications” mean to you? (e.g., does it include over-the-counter and prescribed medications?)*

*Have you ever taken a medication that affected your balance? If so, what did it feel like?*

*Let’s say you tripped last week after you had taken cough syrup. Would you have checked “alcohol and drugs,” “medications,” neither, or both?*

**Impact location**

*Now, I’d like to ask you about the question related to the parts of your body that may have hit the ground or floor during a fall or near-fall.*

*How did you respond to question #23, “What part(s)å of your body hit the ground, floor, surface, or other object during your fall or near-fall?”*

⬜ Head or neck ⬜ Trunk or torso

⬜ Shoulder ⬜ Upper arm

⬜ Elbow ⬜ Lower arm

⬜ Wrist or hand ⬜ Hip or buttocks

⬜ Thigh ⬜ Knee

⬜ Calf ⬜ Ankle or Foot

⬜ Do not remember ⬜ None of these apply

*Are their other body locations that you would expect to hit the floor or ground during a fall that are not listed above? If so, which ones?*

*Do trunk and torso mean the same thing to you? Why?*

*Did you have trouble visualizing any of the body parts listed above? If so, which ones? Why?*

*Can you remember a time when you fell and hit a part of your body where it would be hard to respond to this question with the answers listed? If so, what happened?*

**Medical treatment sought**

*Now, I’d like to ask you about the question related to medical treatment after a fall or near-fall.*

*How did you respond to question #25, “Did you seek treatment from a medical facility because of your fall or near-fall?”*

⬜ No treatment sought

⬜ Primary care clinic

⬜ Urgent care clinic

⬜ Emergency room

⬜ Do not remember

⬜ None of these apply

*Can you describe the difference between primary care and urgent care? Between urgent care and an emergency room?*

*What types of fall-related injuries might to cause someone to go to a primary care clinic? An urgent care clinic? An emergency room?*

*What type of things would you consider in order decide whether you should seek medical attention after a fall-related injury?*

*Are there situations where you would seek medical treatment after a fall or near-fall, but not go to a primary care clinic, urgent care clinic, or an emergency room? If so, where would you go?*

**Questions for Cognitive Interview Group 5**

**Location at the time of the event**

*Now, I’d like to ask you about the question related to your location at the time of the fall or near-fall.*

*How did you respond to question #11, “Were you inside or outside?” (Choose the best answer)*

⬜ Inside ⬜ Outside ⬜ None of these apply

⬜ Do not remember

*What did you think about when you read the terms inside or outside?*

Can you think of any locations that might be hard to describe as inside or outside?

*Can you describe a situation where you were concerned that you might fall because of the location (i.e., indoor or outdoor)?*

*How do you think being inside or outside changes the likelihood that someone may fall?*

**Possible answers**

*Now I would like to ask you questions about each of the possible answers. Even though you may not have checked a box, I’d like you to try to think about situations where these locations might cause someone to lose their balance or fall.*

**Inside**

*Are there fall hazards that are unique to indoor locations? If so, what are they?*

**Outside**

*Are there fall hazards that are unique to outdoor locations? If so, what are they?*

**Ground or floor**

*Now, I’d like to ask you about the question related to the ground or floor at the time of the fall or near-fall. How did you respond to question #14, “What was the ground or floor like?”*

⬜ Flat ⬜ Uneven

⬜ Smooth ⬜ Rough

⬜ Wet ⬜ Dry

⬜ Slippery ⬜ Sloped

⬜ Do not remember ⬜ None of these apply

*Do the words ground and floor mean something different to you? If so, how do they differ?*

*Have we missed any descriptions of ground or floor conditions that may contribute to someone losing his or her balance?*

*Do any of the answers seem repetitive? If so, which ones?*

*Was there a ground or floor condition that you would* ***not*** *expect to contribute to a loss of balance? If so, which one(s)?*

**Possible answers**

*Now I would like to ask you questions about each of the possible answers. Even though you may not have checked a box, I’d like you to try to think about situations where these ground or floor conditions might cause someone to lose their balance or fall.*

**Flat**

*What are some examples of where the ground or floor may be flat, but someone could loss his or her balance and possibly fall?*

*Would you differentiate between a flat and a smooth surface? If so, how? Are they likely to affect someone’s balance the same way?*

*Can you remember a time when you experienced a fall or near-fall on a flat surface? If so, what happened?*

**Smooth**

*Can you describe a few examples of what you would consider to be smooth ground or flooring?*

*Are their any other words you would use to describe a smooth floor? If so, what are they?*

*How concerned would you be about falling if you were walking on a smooth surface? How come?*

**Rough**

*Would you consider rough ground or flooring to be limited to certain locations or environments? If so, which?*

*How do you think rough ground or flooring might cause someone to lose his or her balance and fall?*

*What are some types of rough ground that you would consider likely to cause someone to lose his or her balance?*

**Uneven**

*Are uneven and rough ground different? If so, how? Are they likely to cause someone to fall in the same way?*

*Has there ever been a time when you had a fall or near-fall on uneven ground? If so, can you describe it?*

**Wet**

*Is a wet floor likely to result in a specific direction of fall? If so, can you describe it?*

*Are there certain types of wet floors that someone wearing a prosthesis might be more likely to fall? Why?*

**Slippery**

*Would you consider a slippery floor and a wet floor to be different? If so how?*

*Can you give me a few examples of slippery floors that might cause someone who uses a prosthesis to fall?*

**Dry**

*Do you think a dry floor might cause someone to lose his or her balance and fall? If so, how?*

*Can you give me a few examples of dry floors that might cause someone who uses a prosthesis to fall?*

**Sloped**

*How would you describe a sloped surface?*

*How does standing or walking on a sloped surface affect your balance?*

*Can you give me a few examples of sloped surfaces that might cause someone who uses a prosthesis to fall?*

*How would you have filled out this section if uneven ground were presented as a one-off disruption (e.g., curb) versus a continuous disruption (e.g., gravel pathway)?*

**Catching yourself**

*Now, I’d like to ask you about the question related to what you might have fallen on or caught yourself on at the time of the fall or near-fall.*

*How did you respond to question #22, “Did you fall or catch yourself on or against any of the following?”*

⬜ Ground or floor ⬜ An object

⬜ Another person ⬜ Something else

⬜ Do not remember ⬜ None of these apply

*Is catching yourself on or against something or someone an effective way that you or other LLP users try to avoid a fall? Why or why not?*

*Does catching yourself on another object differ from catching yourself on another person? If so, how?*

*Does the ground or floor, as well as objects and people around you affect what you do when you lose your balance and/or fall? If so, how?*

**Possible answers**

*Now I would like to ask you questions about each of the possible answers. Even though you may not have checked a box, I’d like you to try to think about situations where someone might fall or catch him or herself on.*

**Ground or floor**

*What are some types of ground or floor that you might fall on?*

*Can you catch your self on the ground or floor without falling?*

*Can you describe the ground or floor the last time you fell?*

**An object**

*What types of objects have you grabbed onto in order to avoid falling? What are they?*

*What types of objects have you fallen onto? What are they?*

**Another person**

*Would you be more or less confident in falling or catching yourself against a person or an object? Why or why not?*

*If you were to lose your balance, how would you grab onto another person to avoid falling onto the ground or floor?*

**Something else**

*What other kinds of things might someone fall against or catch them self on when they lose their balance?*

*If you lost your balance and landed on a counter or against the wall or another person, would you consider that a fall or a near-fall? Why?*

**Medical treatment received**

*Now, I’d like to ask you about the question related to medical treatment at the time of the fall or near-fall.*

*How did you respond to question #26, “Did you receive medical treatment because of your fall or near-fall?”*

⬜ Treated and discharged the same day

⬜ Admitted and hospitalized for 1 night

⬜ Admitted and hospitalized for 2-3 nights

⬜ Admitted and hospitalized for more than 3 nights

⬜ Don’t remember

⬜ None of these apply

*What kinds of things does “medical treatment” cover?*

*Have you ever received medical treatment for a fall or near-fall? If so, can you describe what it consisted of?*

*Are being hospitalized and being admitted the same thing? If not, how do they differ?*
